# Isoniazid- and rifampicin-induced thrombocytopenia

**DOI:** 10.1186/2049-6958-8-13

**Published:** 2013-02-13

**Authors:** Fatih Yakar, Namşan Yildiz, Aysun Yakar, Zeki Kılıçaslan

**Affiliations:** 1 Department of Pulmonary Medicine, Bezmialem Vakif University, 34093, Fatih, Istanbul, Turkey; 2 Department of Pulmonary Medicine, IstanbulUniversity, Istanbul Medical Faculty, Istanbul, Turkey; 3 Department of Pulmonary Medicine, Malatya State Hospital, Malatya, Turkey

**Keywords:** Drug toxicity, Thrombocytopenia, Tuberculosis

## Abstract

Treatment of tuberculosis has many side effects. Thrombocytopenia is a serious side effect of such treatment and occurs mostly due to rifampicin (RIF). There are very few reported cases of thrombocytopenia due to isoniazid (INH). An 18-year-old female patient was diagnosed with smear-positive pulmonary tuberculosis. A four-drug regimen [INH, RIF, ethambutol (EMB), and pyrazinamide (PZA)] was given. After the development of thrombocytopenia, the drug treatment was stopped, and a thrombocyte suspension was given until a normal thrombocyte count was obtained. After several start-stop trials, first INH and then RIF were identified as the possible causes of thrombocytopenia and were removed from the regimen. The patient was treated with EMB, streptomycin, PZA, and moxifloxacin with no further development of thrombocytopenia. The current case shows that antituberculosis drugs other than RIF and PZA may be responsible for the development of thrombocytopenia.

## Background

Tuberculosis is a chronic granulomatous infection that has caused mortality and morbidity for centuries. Treatment of this disease is problematic because of its long duration and compliance problems. One of the most important factors causing low compliance is the adverse effects of the drugs. Some of them are self-limiting, but some require treatment cessation. Common adverse effects are hepatotoxicity, hypersensitivity reactions, loss of vision, loss of hearing, flu-like syndrome, hemolytic anemia, acute renal failure, shock, neuropathy, arthralgia, and thrombocytopenia [1]. Although rare, severe thrombocytopenia may be life-threatening. Here we describe the case of a patient affected with thrombocytopenia caused by isoniazid (INH) and rifampicin (RIF).

## Case presentation

An 18-year-old female with no known disease history was admitted to another center with complaints of coughing with sputum and weight loss. Chest x-ray and microscopic examination of the sputum enabled a diagnosis of smear-positive pulmonary tuberculosis. A four-drug regimen (INH, 300 mg/day; RIF, 450 mg/day; ethambutol [EMB], 1,250 mg/day; and pyrazinamide [PZA], 1,500 mg/day) was started according to her weight of 50 kg. While normal at the start of therapy (300,000/mm^3^), her thrombocyte count began to decrease during the second day of treatment (18,000/mm^3^). The therapy was stopped, and after transfusions of fresh frozen plasma and a thrombocyte suspension, her thrombocyte count became normal. Rifampicin was assumed to be the causative factor of thrombocytopenia, and therapy was restarted with INH, PZA, and EMB. However, thrombocytopenia relapsed during this regimen. The treatment was stopped and the patient was referred to our hospital.

When the patient was admitted to our hospital, her main complaint was coughing. No important previous history was present. Physical examination findings were normal with the exception of bilateral ecchymoses on her lower extremities. Chest X-ray showed a cavity with peripheral infiltration in the left upper zone. Laboratory findings revealed; sedimentation rate: 46 mm/hr, CRP: 24 mg/lt, WBC: 9,910/mm^3^, hemoglobin: 10 gr/dl, hematocrit: 31%, and thrombocytes: 135,000/mm^3^. Other parameters were noncontributory.

Because of the recurrent thrombocytopenia, we planned to start a treatment with a single agent and add the others one by one. The treatment was initiated in our hospital with INH at 300 mg/day, but this resulted in thrombocytopenia (30,000/mm^3^) on the third day, and the therapy was stopped. As soon as the thrombocyte count became normal, we began a three-drug regimen according to the patient’s weight: PZA, 1,500 mg/day; streptomycin (SM), 750 mg/day; and EMB, 1,250 mg/day. Because the thrombocyte count was normal (225,000/mm^3^), INH was again added to the regimen, but on the second day of INH initiation, the thrombocyte count decreased to 10,000/mm^3^. The INH was stopped and the patient underwent a consultation in the Hematology Department. Then a thrombocyte suspension and methylprednisolone (1 mg/kg/day) were given. During the follow up, the thrombocyte count became normal and the methylprednisolone therapy was stopped. After re-initiation of the three-drug regimen (EMB, PZA, and SM), RIF at 450 mg/day was added to the therapy as the fourth drug. On the third day of RIF therapy, the thrombocyte count decreased to 34,000/mm^3^ and RIF was ceased.

Because of thrombocytopenia caused by both INH and RIF, moxifloxacin at 400 mg/day was added to the treatment regimen. Without INH and RIF, patient had no further thrombocytopenia development during the period of therapy. After the first month of the therapy, her sputum smear was negative. There were no clinical or laboratory abnormalities during follow up examinations. After cessation of SM in the second month, the antituberculosis treatment regimen was continued and completed with three drugs (EMB, 1250 mg/day; PZA, 1500 mg/day; and moxifloxacin, 400 mg/day) without any further complications.

## Discussion and conclusions

Although there are reports of thrombocytopenia during antituberculosis treatment, only few case reports of INH-induced thrombocytopenia are reported [2-5], but none where the hematologic disorder was at the same time caused by INH and RIF. Our patient had recurrent episodes of thrombocytopenia due to INH and RIF, either together or separately.

A thrombocyte count of <150,000/mm^3^ is defined as thrombocytopenia. However, 2.5% of the normal population may have thrombocyte levels lower than this value [6]. The main mechanisms of thrombocytopenia are decreased production or increased destruction. Moreover, dilutional and distributional mechanisms may contribute. Before diagnosing thrombocytopenia, pseudothrombocytopenia, which may occur due to ineffective anticoagulation in blood tubes, thrombocyte clustering, or abciximab use, should be excluded [7]. In our patient, pseudothrombocytopenia was excluded with a peripheral blood smear examination.

Drug-induced thrombocytopenia is a frequent condition. George *et al.* collected case reports of drug-induced thrombocytopenia and defined the standard criteria with whom to explain the association between the drugs and thrombocytopenia [8]. They defined four criteria: 1) the suspected drug preceded thrombocytopenia and recovery was complete and sustained after the drug withdrawal 2) The suspected drug was the only drug used prior to the onset, or other drugs were continued or reintroduced after discontinuation of the suspected drug with a sustained normal thrombocyte count. 3) Other etiologies of thrombocytopenia were excluded. 4) Re-exposure to the suspected drug resulted in recurrent thrombocytopenia. If the suspected drug meets all criteria, then the level of evidence is definite. If it meets the first three, it is probable; if it meets only the first criterion, it is possible; and if the first criterion is not met, then it is unlikely that it is the responsible agent. According to these criteria, both INH and RIF in our patient met all of the criteria, and so the level of evidence was definite.

Clinical findings usually appear when the thrombocyte count decreases under a certain level. When thrombocytes are lower than 20,000/mm^3^, spontaneous bleeding and ecchymoses may appear. There were ecchymoses on both lower extremities of the present patient **at** admittance. RIF is the most common cause of thrombocytopenia among antituberculosis drugs. The drug binds noncovalently to membrane glycoproteins to produce compound epitopes or induce conformational changes for which antibodies are specific [8]. In addition, RIF-dependent antibodies attach to thrombocytes and cause increased destruction.

INH is a synthetic chemical and a pyridine derivative of nicotinamide. The central nervous system, liver, and hematological system are the main targets of INH toxicity. It may induce acute or chronic disturbances of the hematological system. Acutely it may cause leukocytosis, and chronically it may determine anemia (hemolytic, sideroblastic, aplastic, or megaloblastic), agranulocytosis, eosinophilia, or thrombocytopenia; disseminated intravascular coagulation and lymphadenopathy due to hypersensitivity reactions have also been reported [9]. The exact mechanism of INH-induced thrombocytopenia is not known.

Although INH-induced thrombocytopenia has been defined previously, there are only four previous cases in the literature, none of which was also RIF-induced. INH- and RIF-induced thrombocytopenia in the present case was diagnosed by the occurrence of thrombocytopenia due to RIF in the treatment regimen without INH as well as thrombocytopenia due to INH in the regimen without RIF and exclusion of other potential diagnoses by a peripheral blood smear. Nevertheless, thrombocytopenia is rare but life-threatening. Early recognition and cessation of treatment may prevent mortality and morbidity.

## Consent

Written informed consent was obtained from the patient for publication of this report and any accompanying images.

## Competing interests

The authors declare that they have no competing interests.

## References

[B1] Blumberg HM Burman WJ Chaisson RE Daley CL Etkind SC Friedman LN Fujiwara P Grzemska M Hopewell PC Iseman MD Jasmer RM Koppaka V Menzies RI O'Brien RJ Reves RR Reichman LB Simone PM Starke JR Vernon AA American Thoracic Society, Centers for Disease Control and Prevention and the Infectious Diseases Society American thoracic society/centers for disease control and prevention/infectious diseases society of America: treatment of tuberculosis Am J Respir Crit Care Med 2003 167 603 662 12588714 10.1164/rccm.167.4.603

[B2] Guy C Broyet C Albengres E Berthoux F Ollagnier M Thrombopenia caused by isoniazid Therapie 1993 48 5 490 491 8146836

[B3] Schlegel H Sadoun D Le Roux G Guillevin L Battesti JP Thrombopenia induced by isoniazid Ann Med Interne (Paris) 1991 142 8 630 1807185

[B4] Hansen JE Hypersensitivity to isoniazid with neutropenia and thrombocytopenia Am Rev Respir Dis 1961 83 744 747 13711337 10.1164/arrd.1961.83.5.744

[B5] Laub DR Jr Isoniazid causing drug-induced thrombocytopenia Eplasty 2011 11 ic10 21776327 PMC3121230

[B6] Buckley MF James JW Brown DE Whyte GS Dean MG Chesterman CN Donald JA A novel approach to the assessment of variations in the human platelet count Thromb Haemost 2000 83 3 480 484 10744157

[B7] Landaw SA George JN ***Approach to the adult patient with thrombocytopenia*** UpToDate version 19.3. Last topic update 27 September, 2011 2011 http://www.uptodate.com/contents/approach-to-the-adult-patient-with-thrombocytopenia

[B8] George JN Raskob GE Shah SR Rizvi MA Hamilton SA Osborne S Vondracek T Drug induced thrombocytopenia a systematic review of published case reports Ann Intern Med 1998 129 886 890 10.7326/0003-4819-129-11_Part_1-199812010-00009 9867731

[B9] Isoniazid drug information International Programme on Chemical Safety, Poisons Information Monograph 288 http://www.inchem.org/documents/pims/pharm/pim288.htm. Last accessed February 11, 2013

